# Dynamic changes in the endophytic bacterial community during maturation of *Amorphophallus muelleri* seeds

**DOI:** 10.3389/fmicb.2022.996854

**Published:** 2022-09-26

**Authors:** Min Yang, Ying Qi, Jiani Liu, Zhixing Wu, Penghua Gao, Zebin Chen, Feiyan Huang, Lei Yu

**Affiliations:** ^1^Yunnan Urban Agricultural Engineering and Technological Research Center, College of Agronomy, Kunming University, Kunming, China; ^2^College of Plant Protection, Yunnan Agricultural University, Kunming, China

**Keywords:** *Amorphophallus muelleri*, seeds, maturation, endophytic bacterial community, dynamic

## Abstract

The seed microbiota is considered to be the starting point of the accumulation of plant microbiota, which is conducive to the preservation and germination of seeds and the establishment and development of seedlings. Our understanding of the colonization and migration dynamics of microbial taxa during seed development and maturation is still limited. This study used 16S rRNA high-throughput sequencing to investigate the dynamic changes in the composition and diversity of the endophytic bacterial community during maturation of *Amorphophallus muelleri* seeds. The results showed that as seeds matured (green to red), the Shannon index of their endophytic bacterial community first decreased and then increased, and the ACE and Chao1 indices of the endophytic bacterial community decreased gradually. The Shannon, ACE, and Chao1 indices of the endophytic bacterial community in the seed coat first decreased and then increased. Principal coordinate analysis of the bacterial communities revealed that the seed coat at different maturity stages showed significantly distinct bacterial communities and formed different clusters according to maturity stage. The bacterial communities of green and red seeds showed a clear separation, but they both overlapped with those of yellow seeds, indicating that some core taxa were present throughout seed maturation, but their relative abundance was dynamically changing. As the seeds grew more mature, the relative abundance of some bacterial communities with plant growth-promoting traits and others correlated with plant resistance (e.g., *Burkholderia-Caballeronia-Paraburkholderia*, *Bacillus*, *Pseudomonas*, *Bradyrhizobium*, *Streptomyces*) tended to increase and peaked in fully mature seeds and seed coats. The endophytic bacterial community of *A. muelleri* seeds seems to be driven by the seed maturation state, which can provide a theoretical basis for a comprehensive understanding of the assembly process of the microbial community during *A. muelleri* seed maturation.

## Introduction

Plant endophytes are microorganisms that colonize the internal tissues of plants and are often associated with plants (Yang et al., 2017). Plant endophytes have been found in all plant parts, including roots, stems, leaves, fruits, and seeds (Kumar et al., 2021). They play an important role in each stage of plant development and in plant adaptation to various ecological conditions, making them important to plant growth and health (Márquez et al., 2007; Llorens et al., 2019). There are two main sources of plant endophytes. One is the external environment of the plant surface, and the other is the seed (Liu et al., 2020). Seed-borne endophytes (bacteria, fungi) are particularly important because they are transmitted between successive plant generations through vertical transmission (Shade et al., 2017), and seed endophytes passed down across generations have a profound impact on plant health, quality, productivity, and microecology (Truyens et al., 2015; Nelson, 2018). Among them, seed endophytic bacteria have an especially great impact on plant development. Due to positional advantages, endophytic bacteria in seeds may affect plant growth and adaptability from seed germination to seedling formation and continue to affect plant development over time (Kumar et al., 2021). Various seed-borne endophytic bacteria found in plant tissues use direct or indirect mechanisms to improve plant growth and development and enhance plant tolerance to biotic and abiotic stresses (Santoyo et al., 2016; Shahzad et al., 2018). Specifically, they can promote germination and plant growth by producing auxins and ethylene, mobilizing various nutrients (N, P, K, etc.), and producing siderophores (Maheshwari et al., 2019; Kumar et al., 2020, 2021; Chang et al., 2021); they produce antifungal compounds, toxins, or hydrolytic enzymes to inhibit different plant pathogens to protect plants (Verma et al., 2019); and they indirectly increase plant adaptability by inducing or regulating the expression of plant genes related to growth, development, and defense (Irizarry and White, 2018; Kumar et al., 2021). Díaz Herrera et al. (2016) reported that the endophytes *Paenibacillus* sp., *Pantoea* sp., and *Bacillus* sp. isolated from wheat seeds significantly enhanced plant growth and resistance to *Fusarium graminearum*. Shahzad et al. (2017) reported that various organic acids produced by the seed-borne endophyte *Bacillus amyloliquefaciens* significantly inhibited the growth of pathogenic *Fusarium oxysporum in vitro* and induced systemic resistance to *F. oxysporum* in tomato plants. These studies have accurately and comprehensively demonstrated the important role of seed endophytic bacteria in plant growth and defense, but our understanding of seed-related microbial communities remains insufficient.

Seeds of many crops, including rice (Walitang et al., 2018), maize (Liu et al., 2020), barley (Bziuk et al., 2021), wheat (Kuźniar et al., 2020), and cotton (Irizarry and White, 2017), contain endophytic bacteria. However, endophytic bacteria are present in different parts of the seeds of different plants, and they are affected by the physiological changes in plants caused by biotic and abiotic factors (Shade et al., 2017). Several studies on rice endophytes have shown that endophytes in rice seeds can spread and respond to changes in the external environment and the growth conditions of the host plants (Hardoim et al., 2012). In addition, changes in seed maturation state can lead to changes in the composition of active bacteria (Okunishi et al., 2005). Endophytic bacteria from seeds of different plant species and their effects on plant growth have been extensively studied (Shahzad et al., 2018; Sánchez-López et al., 2018), but few studies have described the existence and role of endophytic bacteria in seed maturation or have investigated their colonization mechanisms during seed maturation (Chesneau et al., 2020). The dynamics of the endophytic bacterial community during seed maturation are still unclear. To our knowledge, this is the first report to elaborate the colonization and dynamics of endophytic bacterial communities during the maturation of *Amorphophallus muelleri* seeds.

Konjac is an economically important crop in southwestern China. It can accumulate a large amount of glucomannan (KGM) and is used in the food, chemical, and pharmaceutical industries (Behera and Ray, 2016). *A. muelleri* is a special commercial konjac cultivar that can reproduce from seeds (triploid asexual reproduction, 2n = 39) and can produce mature seeds without pollination when flowering under artificially induced or natural conditions (the underground bulb grows for 3–5 years). The seeds of *A. muelleri* mature from December to January in an 8-month growth cycle, and each spike generally produces 300–900 seeds. This high reproductive rate in *A. muelleri* is due to its unique seed production mode involving triploid asexual reproduction, providing a far greater reproductive rate than those (4–6 seeds) of the traditional konjac cultivars (Zhao et al., 2013). This seed production mode has been applied on a large scale to konjac production in southwestern China.

We hypothesized that the seeds of *A. muelleri* are inhabited by endophytic bacteria, which have different key functions throughout seed maturation and seedling establishment. We used 16S rRNA high-throughput sequencing to compare the composition and diversity of the endophytic bacterial community in seeds of the same *A. muelleri* plant at different stages of maturity. Then, the bacterial community functions were further predicted by Phylogenetic Investigation of Communities by Reconstruction of Unobserved States (PICRUSt). Our findings can provide new insights into the dynamics of endophytic bacterial community colonization during *A. muelleri* seed maturation, which may serve as a theoretical reference for functional research on and utilization of endophytic bacteria in *A. muelleri* seeds.

## Materials and methods

### Seed collection and surface disinfection

**FIGURE 1:**
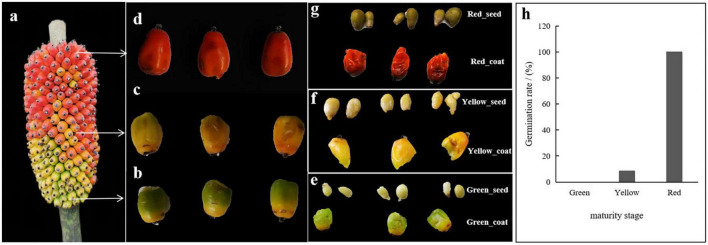
**(a)** *Amorphophallus muelleri* seed-bearing plant. **(b)** Immature-stage seeds (green). **(c)** Intermediate-stage seeds (yellow). **(d)** Fully mature seeds (red). **(e)** Separated green external coat and internal seed. **(f)** Separated yellow external coat and internal seed. **(g)** Separated red external coat and internal seed. **(h)** Germination rate of seeds with different maturation durations (green seeds: 0%; yellow seeds: 8.33%; red seeds: 100%).

Seed samples of *A. muelleri* were collected in the laboratory of Kunming University/Yunnan Urban Agricultural Engineering & Technological Research Center from December 2021 to January 2022. The *A. muelleri* plants were grown in this laboratory (natural environment temperature: 25°C; humidity: 75%) before and during the whole seed sampling process (Figure 1a). The *A. muelleri* seeds were divided into three stages according to their maturity: the immature stage (the seed coat was emerald green, Figure 1b), the intermediate stage (the seed coat gradually changed from emerald green to golden yellow, Figure 1c), and the fully mature stage (the seed coat was bright red, Figure 1d). At different stages of maturity, round and plump seeds were selected as experimental materials, with three duplicates for each maturity stage, and each replicate contained three seeds.

To avoid bacterial contamination of the environment, surface sterilization of collected seeds was performed according to Wang et al. (2021) with some improvements. First, the seeds were rinsed with 50 mL of sterilized distilled water at least three times or until no turbidity was observed. Second, the washed seeds were soaked in 75% ethanol for 30 s and then rinsed with sterilized distilled water three times. Third, the seeds were soaked in 3% sodium hypochlorite solution for 3 min and then rinsed with sterilized distilled water 3 times. Then, 150 μL of water from the last wash was spread on a potato dextrose agar plate, and the plate was placed in a thermostatic incubator at 28°C for 72 h to determine whether the seed surface was completely sterilized (each seed was examined three times). Finally, the external seed coat and the internal seed were separated using a sterile scalpel on an ultraclean workbench (Figures 1e–g). To remove the bacteria on the surface and prevent cross-contamination of the bacteria that were in the seed coats and seeds, the above sterilization steps were repeated for the separated seed coats and seeds. The samples were placed in a 10-mL sterile conical tube and immediately stored in a -80°C freezer after quick freezing in liquid nitrogen for later use. The experiment included a total of six treatments, which were named Green_seed, Green_coat, Yellow_seed, Yellow_coat, Red_seed, and Red_coat. Each treatment had three replicates (each replicate contained three seeds, and three seeds were ground with an autoclaved mortar and pestle as a sample), for a total of 18 samples.

### DNA extraction and polymerase chain reaction amplification, and sequencing

The total DNA of each sample (sample of grinding with 3 seeds, 0.5 g) was extracted according to the procedure of the FastDNA^®^SPIN Kit for Soil (MP, USA). The DNA concentration and purity were determined using a NanoDrop2000 instrument. The DNA extraction quality was determined by 1% agarose gel electrophoresis.

In the analysis of bacterial communities, two-step polymerase chain reaction (PCR) amplification was performed. First, the 16S rRNA gene was amplified using bacterial primers 799F (5′-AACMGGATTAGATACCCKG-3′) and 1392R (5′-ACGGGCGGTGTGTRC-3′) (Hanshew et al., 2013). The 20-μL amplification system was prepared by mixing 4 μL 5 × FastPfu Buffer, 2 μL 2.5 mM dNTPs, 0.8 μL forward primer (5 μM), 0.8 μL reverse primer (5 μM), 0.4 μL FastPfu Polymerase, 0.2 μL BSA, 10 ng template DNA, and ddH_2_O to 20 μL. The amplification program was as follows: predenaturation at 95°C for 3 min, followed by 27 cycles of thermal cycling (denaturation at 95°C for 30 s, annealing at 55°C for 30 s, extension at 72°C for 45 s) and a final extension at 72°C for 10 min. The primers used in the second PCR amplification were 799F (5′-AACMGGATTAGATACCCKG-3′) and 1193R (5′-ACGTCATCCCCACCTTCC-3′) (Bulgarelli et al., 2012). All the conditions of this PCR step were the same as those of the first PCR amplification except that only 13 cycles of thermal cycling were performed. The PCR products were recovered from a 2% agarose gel and further purified using the AxyPrep DNA Gel Extraction Kit (Axygen Biosciences, Union City, CA, USA) according to the manufacturer’s instructions. The PCR amplicons were sequenced on the Illumina MiSeq PE300 platform at Shanghai Majorbio Bio-Pharm Technology Co., Ltd.

### Quality control and analysis of offline data

Trimmomatic software was used for quality control of the original sequencing sequences, and FLASH software (version 1.2.11) was used for sequence assembly (Caporaso et al., 2011). USEARCH software (version 11) (Edgar, 2013) was used to filter the obtained sequences and remove the chimera sequences to obtain valid sequences. UPARSE software (version 11) was used to divide the operational taxonomic units at a similarity level of 97%. RDP classifier software (version 2.13) (Wang et al., 2007) and the SILVA database (version 138) (Altschul et al., 1990) were used for species annotation. Mothur (version 1.30.2) was used to generate the dilution curves; to calculate the library coverage and the Shannon, Simpson, ACE, and Chao1 indices; and to evaluate the species diversity and abundance indices. Principal coordinate analysis (PCoA) was performed using the Bray–Curtis distance algorithm established by QIIME software (version 1.9.1). Permutational multivariate analysis of variance (PERMANOVA) was performed using the adonis function from the vegan package in R to confirm the changes in the bacterial communities (Oksanen et al., 2016). The Circos diagram was drawn with Circos-0.67-7 software (Yan et al., 2020). Linear discriminant analysis (LDA) of effect size (LEfSe) was used to search for significantly different biomarkers between different seed compartments (seed coat and seed) of different maturity stages by using LEfSe software. LEfSe employs the non-parametric factorial Kruskal–Wallis sum-rank test (α = 0.05) to identify taxa with significantly different abundances between categories [using all-against-all (stricter)] followed by LDA to estimate the effect size of each feature with differential abundance (logarithmic LDA score = 2.0). PICRUSt software (version 2.2.0) was used to predict the functions of the endophytic bacterial community in each sample. Data were statistically analyzed using analysis of variance (ANOVA) at *p* < 0.05, and means were compared using least significant difference (LSD) and Duncan’s multiple range test at *p* < 0.05 (Zhang et al., 2020).

## Result analysis

### Diversity of bacterial communities

**FIGURE 2:**
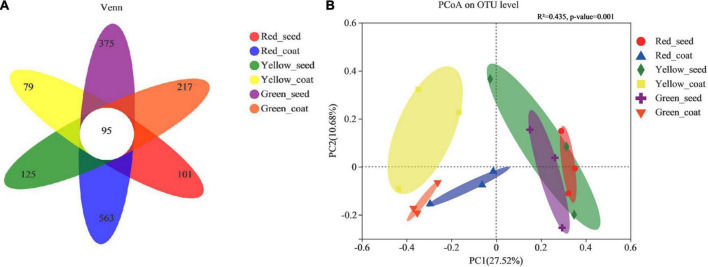
Venn diagram showing the number of unique, shared, and common bacterial operational taxonomic units. **(A)** Principal coordinate analysis (PCoA) plots based on the Bray–Curtis dissimilarity matrix showing the changes in the structure of bacterial communities. **(B)** (PERMANOVA, *R*^2^ = 0.435, *p* < 0.001).

The 16S rRNA genes of seeds and seed coats of different maturity stages were sequenced, and the coverage of all samples was greater than 99.95% (Table 1), indicating that the sequencing information was sufficient to reveal the majority of bacterial communities in each sample. A total of 995,942 effective bacterial sequences were read from the 18 samples, which were divided into 2397 bacterial operational taxonomic units (OTUs). The 2397 bacterial OTUs were matched to 37 different phyla, 845 genera, and 1453 species. The number of OTUs (97% clustering level) and the diversity and abundance indices of different samples are shown in Table 1. When the seeds and seed coats of different treatments were compared, the number of bacterial OTUs was ranked as follows: Green_seed > Green_coat, Yellow_seed > Yellow_coat, and Red_coat > Red_seed. The comparison of seeds at different maturity stages showed that the number of bacterial OTUs in the seeds decreased with increasing seed maturity, i.e., Red_seed < Yellow_seed < Green_seed. The Venn diagram of bacterial OTUs (Figure 2A) showed that all samples shared only 95 common OTUs and that the number of OTUs unique to each sample was ranked as follows: Red_coat (563) > Green_seed (375) > Green_coat (217) > Yellow_seed (125) > Red_seed (101) > Yellow_coat (79).

**TABLE 1:** Alpha diversity indices in each treatment (± SEM, *n* = 3/treatment).

Sample name	Sequence numbers	Coverage/%	Number of OTUs	Alpha diversity			
				
				Shannon	Simpson	ACE	Chao1
Red_seed	52200	99.97 ± 0.007	526	4.36 ± 0.18a	0.0301 ± 0.0086b	266.55 ± 33.73a	258.09 ± 36.40a
Red_coat	57668	99.96 ± 0.013	1117	4.09 ± 0.89a	0.0797 ± 0.0458ab	639.57 ± 238.52a	504.30 ± 242.19a
Yellow_seed	54276	99.95 ± 0.019	679	4.22 ± 0.54a	0.0468 ± 0.0188b	353.05 ± 93.61a	343.14 ± 103.55a
Yellow_coat	53715	99.96 ± 0.013	389	2.55 ± 0.71a	0.2307 ± 0.0913a	195.57 ± 28.49a	194.18 ± 29.39a
Green_seed	57014	99.96 ± 0.009	1096	4.30 ± 0.65a	0.0708 ± 0.0537ab	482.98 ± 250.71a	480.18 ± 252.35a
Green_coat	57109	99.98 ± 0.006	604	3.02 ± 0.56a	0.1672 ± 0.0600ab	277.23 ± 52.42a	276.14 ± 55.15a

Significant differences among treatments are shown by different lowercase letters within the columns according to a least significant difference test (LSD; p < 0.05).

Similar to the number of OTUs, the bacterial diversity and abundance indices also differed among samples. When comparing seeds and seed coats of different treatments, the Shannon index of the endophytic bacterial community was larger in seeds than in seed coats at each maturity stage, i.e., Green_seed > Green_coat, Yellow_seed > Yellow_coat, and Red_seed > Red_coat. The ACE and Chao1 indices of the endophytic bacterial community were as follows: Green_seed > Green_coat, Yellow_seed > Yellow_coat, and Red_coat > Red_seed. Further analysis of the maturity-related differences showed that as the seeds grew more mature (green to red), the Shannon index of the endophytic bacterial community in the seeds showed a trend of first decreasing and then increasing, while the ACE and Chao1 indices in the seeds showed a gradual decreasing trend. As the seeds grew more mature, the Shannon, ACE, and Chao1 indices of the endophytic bacterial community in the seed coats all showed a trend of first decreasing and then increasing, whereas no significant difference was observed for the Shannon, ACE, and Chao1 indices (LSD, *p* > 0.05). Overall, the number of OTUs and the diversity and abundance indices of the endophytic bacterial community were different between seeds and seed coats of different maturity stages.

The PCoA was performed by using the Bray–Curtis distance algorithm. Principal component 1 (PCo1) and principal component 2 (PCo2) explained 27.52 and 10.68% of the sample difference, respectively, so the two together could explain 38.20% of the sample difference. Figure 2B shows significant differences in the bacterial community composition between seed coats and seeds in different treatments. The bacterial communities in seed coats were mostly on the left side of the x-axis, while the bacterial communities in seeds were mostly on the right side of the x-axis. The long distance between the two indicated that the bacterial community compositions in seeds and seed coats were evidently different. Further analysis of the differences between different maturity stages showed that the seed coat samples of different maturity stages also showed significantly separated bacterial communities and formed different clusters according to maturity stage, which indicated that the seed coats of different maturity stages had markedly different bacterial community compositions. The bacterial communities in green and red seeds were significantly separated, but they both overlapped with those in yellow seeds. The bacterial community structures of the green and red seeds were significantly different, but they were not significantly different from that of the yellow seeds. The above results indicate that maturity may be an important factor causing microbial community differences.

### Species composition and relative abundance of bacterial communities

**FIGURE 3:**
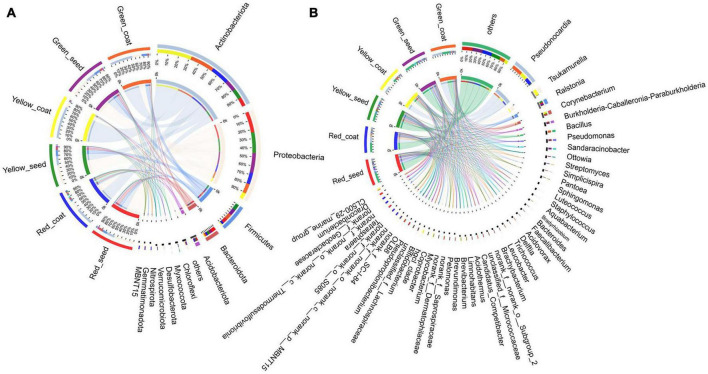
The composition of most dominant endophytic bacterial communities at the phylum **(A)** and genus levels **(B)** under different treatments (*n* = 3/treatment).

The dominant phyla of the endophytic bacterial communities under the different treatments were Actinobacteria, Proteobacteria, Firmicutes, Bacteroidota, and Acidobacteria (Figure 3A and Supplementary Table 1). When comparing the seeds and seed coats of different maturity stages, we found that the abundance of Actinobacteria was higher in seed coats than in seeds at all three maturity stages, while the relative abundances of Proteobacteria, Firmicutes, Bacteroidota, and Acidobacteria were higher in seeds than in seed coats. With the increase in maturity stage (green to red), the relative abundance of Actinobacteria showed a gradual decreasing trend in the seeds and a trend of first increasing and then decreasing in the seed coats; the relative abundances of Proteobacteria and Acidobacteria showed a trend of gradually increasing in seeds and a trend of first decreasing and then increasing in seed coats; and the relative abundances of Firmicutes and Bacteroidota in green seeds was not significantly different from that in yellow seeds, but after the seeds were fully mature (red), the relative abundance of Firmicutes decreased, and the relative abundance of Bacteroidota increased.

**FIGURE 4:**
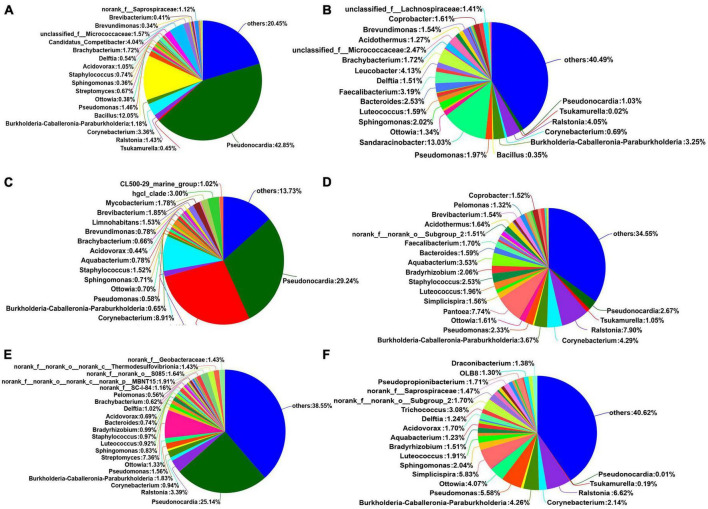
Relative abundances of endophytic bacterial communities in different treatments at the genus level (*n* = 3/treatment). **(A)** Green_coat, **(B)** Green_seed, **(C)** Yellow_coat, **(D)** Yellow_seed, **(E)** Red_coat, **(F)** Red_seed.

The dominant bacterial genera in the six treatments were *Pseudonocardia*, *Tsukamurella*, *Ralstonia*, *Corynebacterium*, and *Burkholderia-Caballeronia-Paraburkholderia* (Figure 3B and Supplementary Table 2). The relative abundance of *Pseudonocardia* was greater in seed coats than in seeds at each maturity stage. As the seeds grew more mature, the relative abundance in seed coats gradually decreased. The relative abundances of *Ralstonia*, *Burkholderia-Caballeronia-Paraburkholderia*, *Pseudomonas*, *Sphingomonas*, *Luteococcus*, *Bradyrhizobium*, *Aquabacter*, *Bacteroides*, *Acidovorax*, *Delftia*, and *Trichococcus* were greater in seeds than in seed coats (Figure 4). With the increase in maturity, the relative abundances of *Burkholderia-Caballeronia-Paraburkholderia*, *Bacillus*, and *Pseudomonas* increased in seeds but first decreased and then increased in seed coats. Some bacterial genera were significantly present in samples in a certain period. For example, the relative abundance of *Tsukamurella* in the Yellow_coat samples (27.94%) was significantly higher than that in other samples (0.02–1.05%); the relative abundance of *Bacillus* in Green_coat samples (12.05%) was significantly higher than that in other samples (0.12–0.65%); the relative abundance of *Sandaracinobacter* in Green_seed samples (13.03%) was significantly higher than that in other samples (0–0.0065%); and the relative abundance of *Streptomyces* in the Red_coat samples (7.36%) was significantly higher than that in other samples (0.07–0.67%) (Figure 4). The above results indicate that maturity has a significant impact on the distribution and composition of the endophytic bacterial community in seeds.

**FIGURE 5:**
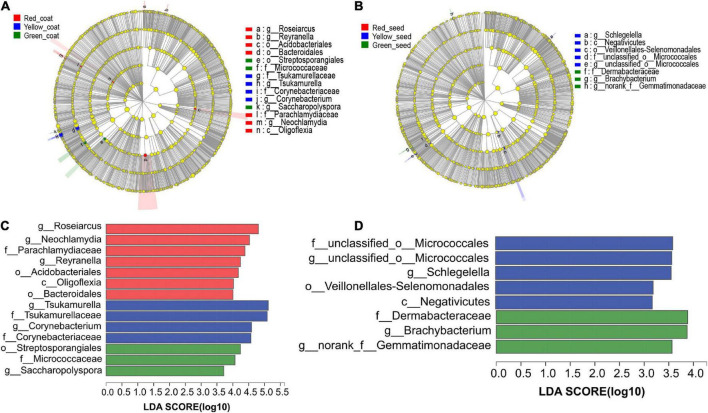
LEfSe analyses of the aggregated groups of bacterial communities in coats and seeds at different maturity stages (taxa from the phylum to the genus level). Cladogram representing the abundance of the taxa in the coats **(A)** and seeds **(B)**. Histogram of the microbiota of coats **(C)** and seeds **(D)** with LDA = 2.

We next determined which taxa of endophytic bacteria distinguished seeds and seed coats of different maturity stages. The LDA of the samples was performed by using LEfSe multilevel species difference analysis according to the taxonomic composition to identify the communities or species that had significantly different influences on sample classification. Figure 5 shows that among the species with an LDA value greater than 2, *Roseiarcus* (genus), *Neochlamydia* (genus), Parachlamydiaceae (family), *Reyranella* (genus), and Acidobacteriales (order) were mainly enriched in the red seed coats; Tsukamurella (family to genus), *Corynebacterium* (genus), and Corynebacteriaceae (family) were mainly enriched in the yellow seed coats; and Streptosporangiales (order), Micrococcaceae (family), and *Saccharopolyspora* (genus) were mainly enriched in the green seed coats. In addition, unclassified_o__Micrococcales (family to genus), *Schlegelella* (genus), Veillonellales_Selenomonadales (order), and Negativicutes (class) were mainly enriched in the yellow seeds, while Dermabacteraceae (family), *Brachybacterium* (genus), and *norank_f__Gemmatimonadaceae* (genus) were mainly enriched in the green seeds. No species were significantly enriched in the red seeds. These microorganisms enriched in different samples may be important taxa that cause differences in community structure.

### Predictive functional analysis of endophytic bacterial communities

**FIGURE 6:**
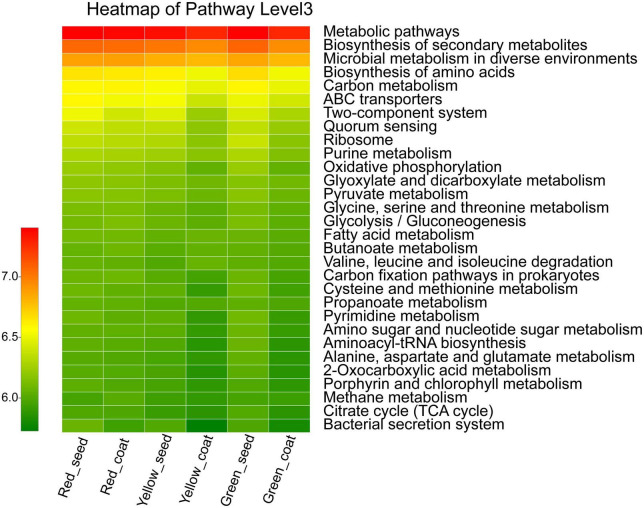
Heatmap of the relative abundances of the top 30 PICRUSt-predicted genes in the different treatments (*n* = 3/treatment). The abscissa is the treatment, and the ordinate is the function name of pathway level 3. The color gradients of different color blocks represent the changes in different functional abundances in the treatments. The legend is the value represented by the color gradient.

The functional characteristics of the endophytic microbial community in *A. muelleri* seeds were predicted using PICRUSt software. PICRUSt identified the 16S rRNA gene sequences based on the Kyoto Encyclopedia of Genes and Genomes pathway database and inferred the possible gene contents of bacteria. Most of the functional genes predicted during the maturation of *A. muelleri* seeds were assigned to functional groups such as metabolism, environmental information processing, genetic information processing, and cellular processes. Among the functions with the top 30 relative abundances at pathway level 3 (Figure 6 and Supplementary Table 3), 80% were metabolism-related functions, 10% were environmental information processing-related functions, and 6.7% were genetic information processing-related functions. Among the environmental information processing-related functions, the ABC transporters, bacterial secretion system, and two-component system showed significant differences between groups (*P* < 0.05), and their relative abundances in seeds and seed coats during seed maturation (green to red) were all ranked in the order Red_seed > Yellow_seed > Green_seed and Red_coat > Green_coat > Yellow_coat. The relative abundances of metabolic-related functions such as biosynthesis of amino acids; carbon metabolism; oxidative phosphorylation; glycine, serine, and threonine metabolism; cysteine and methionine metabolism; and amino sugar and nucleotide sugar metabolism during maturation (green to red) first decreased and then increased. In other words, their relative abundances were the lowest in the intermediate stage (yellow seeds), but the difference between the treatments was not significant.

## Discussion

Endophytic bacteria in seeds are not only closely related to seed activity and seed quality but also affect various seed traits, such as seed germination, plant growth, disease occurrence, and stress resistance (Walitang et al., 2017; Wang et al., 2021). In this study, high-throughput sequencing was used to reveal the composition and diversity of endophytic bacterial communities in *A. muelleri*. Our results showed that as the maturity stage increased (green to red), the Shannon index of seeds showed a trend of first decreasing and then increasing, and the ACE and Chao1 indices showed a gradual decreasing trend; the Shannon, ACE, and Chao1 indices of seed coats all first decreased and then increased. These trends indicate that the endophytic bacterial communities in the seeds and seed coats of *A. muelleri* are dynamic and that the changes in the composition and diversity of endophytic bacterial communities are driven by the seed maturation state. When we analyzed the distribution of endophytic bacterial communities in the seeds and seed coats of *A. muelleri*, PCoA revealed evident separation between the endophytic bacterial communities in seeds and seed coats, further supporting a significantly different endophytic bacterial community composition between seeds and seed coats. More interestingly, the seed coat samples of different maturity stages also showed significantly separated endophytic bacterial communities and formed different clusters according to maturity stage, indicating that the endophytic bacterial community compositions of the seed coats of different maturity stages were different. The endophytic bacterial communities of green and red seeds showed a clear separation, but they both overlapped with those of yellow seeds because the yellow seeds are in the intermediate stage of *A. muelleri* seed maturation.

Endophytes in seeds are passed on from generation to generation, and their core microbiome does not change (Truyens et al., 2015; Shahzad et al., 2018). Chesneau et al. (2020) reported that the bacterial communities in seeds are composed of a small number of dominant taxa, which appear in the early stage of seed filling and persist throughout seed ripening. Thus, it is not surprising that the seed microbiomes at different maturity stages showed a certain degree of overlap. One possible explanation for the apparent separation of endophytic bacterial communities between seed coats of different maturity stages is that this separation is the result of the joint action of seed development and environmental conditions. The seed coat acts as a regulator of the interaction between the seed and the environment, modulating gas exchange and water absorption, and thus is more affected by environmental conditions. Seed coats at different maturity stages may have different responses to the same environment (Dübbern De Souza and Marcos-Filho, 2001; Ma et al., 2004). The above results further show that the maturation state is the main source of variation in the endophytic bacterial community of *A. muelleri*.

We further investigated the dynamics of the endophytic bacterial community during the maturation of *A. muelleri*. The dominant phyla of endophytic bacteria in *A. muelleri* were Actinobacteria, Proteobacteria, Firmicutes, Bacteroidota, and Acidobacteria, which are also dominant phyla in the seed microbiomes of rice (Matsumoto et al., 2021), pumpkin (Adam et al., 2018), and wild cabbage (Tyc et al., 2020). We also detected *Ralstonia*, *Burkholderia-Caballeronia-Paraburkholderia*, *Bacillus*, *Pseudomonas*, *Streptomyces*, *Pantoea*, and *Sphingomonas* in the seeds of different plants by high-frequency separation or identification (Zhang et al., 2018; Liu et al., 2020). Since these phyla and genera are found in the seeds of many plant species (including monocots and dicots), bacteria of these phyla and genera can be considered the universal core seed microbiome. These core microbes will adapt to plant compartments and will persist in seeds, but the relative abundance of the core seed microbiome members may differ due to factors such as plant species, genotype, physiology, developmental stage, and soil type (Bziuk et al., 2021). Our study also found that the relative abundances of endophytic bacterial communities in *A. muelleri* changed dynamically. *Pseudomonas* is a common plant growth-promoting bacterium (PGPB) that may directly or indirectly affect the growth, health, and development of host plants (Liu et al., 2012). *Burkholderia* can produce high concentrations of indole-3-acetic acid and is considered an important endophytic bacterium that directly promotes the growth of related plants (Žiarovská et al., 2020). *Bacillus pumilus* and *Bacillus subtilis* can also help plants to develop faster (Fang and Xiong, 2015). Moreover, the ability to fix atmospheric nitrogen has been found in most PGPBs, including *Bacillus*, *Pseudomonas*, and *Burkholderia* species (Shah et al., 2022). Interestingly, our results showed that as the maturity stage increased, the relative abundances of *Burkholderia*, *Bacillus*, and *Pseudomonas* in *A. muelleri* gradually increased, peaking after the seeds fully matured. Mano et al. (2006) also found that *Bacillus* was more abundant in mature rice seeds. *Bradyrhizobium* is a group of slow-growing, alkali-producing, nitrogen-fixing rhizobia. Some *Bradyrhizobium* strains can fix nitrogen and can promote the growth and root development of some plants (Wang et al., 2021). In this study, with the increase in maturity stage, the relative abundance of *Bradyrhizobium* in the seed coat showed a gradually increasing trend. In addition to promoting plant growth, some endophytic bacteria are associated with plant resistance (antagonistic effects against plant pathogens). For example, seed-borne *Pantoea* and *Paenibacillus* have antifungal effects (Ruiz et al., 2011), a *Burkholderia gladioli* antibiotic has effective activity against *Mycobacterium tuberculosis* (Song et al., 2017), and *Streptomyces* is known for its efficient synthesis of antibiotic compounds that inhibit a variety of plant pathogens (Palaniyandi et al., 2013). In this study, the relative abundance of *Streptomyces* in the red seed coats was significantly higher than that in other samples. The seed coat is the “armor” of the seed, so the significant increase in the relative abundance of *Streptomyces* is conducive to increasing the resistance of the mature seed coat and protecting the seed from the invasion of plant pathogens. Endophytic bacteria can improve plant adaptability by enhancing nutrient mobilization, nitrogen fixation, and phosphate solubilization and conferring plant resistance to pathogens (Hardoim et al., 2012; Wang et al., 2021). Therefore, we speculate that fully mature seeds and seed coats contain many microorganisms with plant growth-promoting traits and contribute to plant resistance, which may contribute to the establishment and development of seedlings of *A. muelleri*. We found that the red seeds had a higher germination rate (Figure 1h).

PICRUSt functional prediction showed that some functions related to membrane transport, signal transduction, and metabolism of the endophytic bacterial community changed dynamically during seed maturation of *A. muelleri*. For example, the relative abundances of membrane transport-related ABC transporters and the bacterial secretion system and the signal transduction-related two-component system in seeds gradually increased with the maturity stage and peaked when the seeds were fully mature. The relative abundances of metabolism-related functions, including amino acid synthesis; carbon metabolism; oxidative phosphorylation; glycine, serine, and threonine metabolism; cysteine and methionine metabolism; and amino sugar and nucleotide sugar metabolism, showed a trend of first decreasing and then increasing in both seeds and seed coats during seed maturation (from green to red). The above results indicate that the maturation state affects the basic life function of the endophytic bacterial community of *A. muelleri* seeds and the process of information transmission from the surrounding environment. At present, the preliminary prediction of the functions of relevant bacteria solely by PICRUSt still has substantial limitations. The functions of bacterial communities during the maturation of *A. muelleri* should be comprehensively analyzed by integrating conventional isolation culture methods and metagenomic methods.

## Conclusion

Our study shows that many endophytic bacterial communities are present in the seeds of *A. muelleri* and that some core taxa are present throughout the seed maturation process but that the diversity of endophytic bacterial communities and the relative abundance of these taxa change dynamically. The relative abundances of some bacterial communities with plant growth-promoting traits and others associated with plant resistance increase (e.g., *Burkholderia-Caballeronia-Paraburkholderia*, *Bacillus*, *Pseudomonas*, *Bradyrhizobium*, *Streptomyces*) with increasing seed maturity and peak in fully mature seeds and seed coats. Endophytic bacteria that were shown to induce plant resistance in previous studies belonged to genera that were highly abundant in the seeds. These microorganisms may contribute to the establishment and development of seedlings of *A. muelleri*. In conclusion, our work supports that the endophytic bacterial community of *A. muelleri* seeds seems to be driven by the seed maturation state. These findings might provide valuable information for a better understanding of the colonization and migration dynamics of microbial taxa during seed development and maturation.

## Data availability statement

The datasets presented in this study can be found in online repositories. The names of the repository/repositories and accession number(s) can be found below: NCBI under accession number: PRJNA862316 (SRA: SRR20646354-SRR20646371).

## Author contributions

PG proposed the idea. YQ and ZC designed and performed the experiments. MY drafted the manuscript. JL and ZW performed the data analyses. LY and FH helped perform the analyses with constructive discussions and revisions of text passages. All authors contributed to the article and approved the submitted version.
