# Supplementary material for: Dynamic changes in the endophytic bacterial community during maturation of Amorphophallus muelleri seeds
Source: Front Microbiol. 2022 Sep 26;13:996854. doi: 10.3389/fmicb.2022.996854 (PMC9549114; doi:10.3389/fmicb.2022.996854)
Supplement: Supplementary file 1 [file Table_1.docx]

| Table S1 The relative abundances of dominant (top-5) endophytic bacterial communities on phylum level at different treatments (±SEM, n = 3/treatment, %) | | | | | | |
| --- | --- | --- | --- | --- | --- | --- |
| Taxonomic category (phylum) | Green_seed | Green_coat | Yellow_seed | Yellow_coat | Red_seed | Red_coat |
|  |  |  |  |  |  |  |
| Actinobacteriota | 24.64±7.25bc | 57.24±12.94ab | 24.14±5.36bc | 79.43±11.38a | 20.22±1.71c | 46.92±16.51abc |
| Proteobacteria | 46.18±11.32a | 22.40±11.81ab | 49.18±9.12a | 14.85±8.73b | 49.83±5.42a | 26.17±6.46ab |
| Firmicutes | 14.97±10.06a | 13.54±11.22a | 14.63±4.60a | 2.87±0.57a | 11.20±1.52a | 5.34±2.49a |
| Bacteroidota | 6.45±3.99ab | 3.75±2.47ab | 5.52±2.68ab | 2.26±1.79b | 10.91±2.25a | 4.52±0.72ab |
| Acidobacteriota | 3.23±2.29a | 0.51±0.15a | 3.42±1.85a | 0.03±0.01a | 3.99±0.42a | 2.15±1.65a |

Significant differences among treatments are shown by different lowercase letters within the row according to a least significant difference test (LSD; p < 0.05).
