# Supplementary material for: Dynamic changes in the endophytic bacterial community during maturation of Amorphophallus muelleri seeds
Source: Front Microbiol. 2022 Sep 26;13:996854. doi: 10.3389/fmicb.2022.996854 (PMC9549114; doi:10.3389/fmicb.2022.996854)
Supplement: Supplementary file 2 [file Table_2.docx]

| Table S2 The relative abundances of dominant (top-5) endophytic bacterial communities on genus level at different treatments (±SEM, n = 3/treatment, %) | | | | | | |
| --- | --- | --- | --- | --- | --- | --- |
| Taxonomic category (genus) | Green_seed | Green_coat | Yellow_seed | Yellow_coat | Red_seed | Red_coat |
|  |  |  |  |  |  |  |
| *Pseudonocardia* | 1.03±0.43b | 42.85±12.64a | 2.67±1.30b | 29.24±17.46ab | 0.01±0.003b | 25.14±10.40ab |
| *Tsukamurella* | 0.02±0.005b | 0.45±0.07b | 1.05±0.70b | 27.94±16.25a | 0.19±0.11b | 0.02±0.00b |
| *Ralstonia* | 4.05±1.62ab | 1.43±1.03ab | 7.9±2.16a | 1.42±0.86ab | 6.62±2.41ab | 3.39±1.68ab |
| *Corynebacterium* | 0.69±0.20c | 3.36±0.22ab | 4.29±2.93ab | 8.91±2.64a | 2.14±0.80c | 0.95±0.50c |
| *Burkholderia*-*Caballeronia*-*Paraburkholderia* | 3.25±1.47ab | 1.18±0.21ab | 3.67±1.08ab | 0.65±0.31b | 4.26±1.03a | 1.83±0.98ab |

Significant differences among treatments are shown by different lowercase letters within the row according to a least significant difference test (LSD; p < 0.05).
