# Supplementary material for: Dynamic changes in the endophytic bacterial community during maturation of Amorphophallus muelleri seeds
Source: Front Microbiol. 2022 Sep 26;13:996854. doi: 10.3389/fmicb.2022.996854 (PMC9549114; doi:10.3389/fmicb.2022.996854)
Supplement: Supplementary file 3 [file Table_3.docx]

| Table S3 Relative abundances of the top-30 functions at pathway level 3 in different treatments (n = 3/treatment) | | | | | | | |
| --- | --- | --- | --- | --- | --- | --- | --- |
| Pathway level 3 | Description | Green_seed | Green_coat | Yellow_seed | Yellow_coat | Red_seed | Red_coat |
| ko01100 | Metabolic pathways | 25115975.68 | 19082933.01 | 22818937.75 | 18883671.57 | 25450662.03 | 24350301.19 |
| ko01110 | Biosynthesis of secondary metabolites | 11821368.78 | 8768622.91 | 10412050.82 | 8888568.27 | 11564461.08 | 11335100.23 |
| ko01120 | Microbial metabolism in diverse environments | 7206876.61 | 6194145.09 | 6732861.39 | 6230719.89 | 7490193.43 | 7579799.90 |
| ko01230 | Biosynthesis of amino acids | 4746320.62 | 3323500.68 | 4134518.94 | 3264798.86 | 4559102.74 | 4364829.47 |
| ko01200 | Carbon metabolism | 3934512.43 | 3117148.05 | 3481293.32 | 3020844.58 | 3902392.32 | 3965493.44 |
| ko02010 | ABC transporters | 3203136.61 | 2570437.15 | 3460038.10 | 2443945.76 | 3876071.77 | 3361270.48 |
| ko02020 | Two-component system | 2699893.83 | 1948437.30 | 2871925.63 | 1694092.01 | 3292246.63 | 2531772.51 |
| ko02024 | Quorum sensing | 2243845.33 | 1677151.74 | 2233371.07 | 1534572.05 | 2495381.69 | 2225219.93 |
| ko03010 | Ribosome | 2413869.30 | 1492555.51 | 2004971.63 | 1531973.93 | 2227648.39 | 2107838.29 |
| ko00230 | Purine metabolism | 1979563.16 | 1410662.26 | 1781378.62 | 1499962.64 | 1960406.90 | 1853734.32 |
| ko00190 | Oxidative phosphorylation | 1681160.81 | 1128568.96 | 1443108.15 | 1114300.29 | 1702433.21 | 1577476.24 |
| ko00630 | Glyoxylate and dicarboxylate metabolism | 1454895.43 | 1252760.76 | 1373679.51 | 1231812.17 | 1544697.70 | 1508690.31 |
| ko00620 | Pyruvate metabolism | 1487501.14 | 1196097.55 | 1367370.42 | 1201517.77 | 1526594.38 | 1458032.28 |
| ko00260 | Glycine, serine and threonine metabolism | 1322232.03 | 1103821.32 | 1189053.73 | 1068533.50 | 1349546.71 | 1357547.61 |
| ko00010 | Glycolysis/Gluconeogenesis | 1353535.76 | 1065787.63 | 1201686.42 | 1077589.57 | 1285400.22 | 1368818.02 |
| ko01212 | Fatty acid metabolism | 1175468.65 | 1046785.34 | 1118183.51 | 1169253.28 | 1278222.69 | 1247884.38 |
| ko00650 | Butanoate metabolism | 1120384.06 | 1013860.08 | 1047957.12 | 1119001.00 | 1154692.31 | 1218061.05 |
| ko00280 | Valine, leucine, and isoleucine degradation | 1093989.82 | 1000366.03 | 976133.86 | 1154978.53 | 1162606.03 | 1112497.96 |
| ko00720 | Carbon fixation pathways in prokaryotes | 1183751.83 | 912419.37 | 1038936.46 | 891082.22 | 1189930.75 | 1223728.57 |
| ko00270 | Cysteine and methionine metabolism | 1217547.08 | 873875.90 | 1083255.87 | 801865.66 | 1213977.48 | 1112264.52 |
| ko00640 | Propanoate metabolism | 1094510.54 | 960355.01 | 987675.42 | 998239.21 | 1126147.58 | 1103771.89 |
| ko00240 | Pyrimidine metabolism | 1256320.15 | 807803.24 | 1093923.73 | 779167.82 | 1214520.83 | 1074083.40 |
| ko00520 | Amino sugar and nucleotide sugar metabolism | 1190602.84 | 809487.27 | 1058207.68 | 788742.69 | 1108558.97 | 1099380.89 |
| ko00970 | Aminoacyl-tRNA biosynthesis | 1166627.17 | 737099.86 | 983682.97 | 728071.19 | 1084516.86 | 1037013.26 |
| ko00250 | Alanine, aspartate, and glutamate metabolism | 1115759.81 | 740020.78 | 928779.58 | 770666.75 | 1023244.78 | 984895.33 |
| ko01210 | 2-Oxocarboxylic acid metabolism | 1055292.47 | 725209.03 | 927298.01 | 712069.05 | 1033666.80 | 1001535.94 |
| ko00860 | Porphyrin and chlorophyll metabolism | 975629.97 | 751261.45 | 907217.23 | 751620.91 | 1070485.13 | 976713.95 |
| ko00680 | Methane metabolism | 902947.47 | 834051.00 | 856712.45 | 774614.22 | 906204.58 | 1026176.38 |
| ko00020 | Citrate cycle (TCA cycle) | 988847.28 | 734011.59 | 820328.91 | 732664.35 | 965211.58 | 964387.58 |
| ko03070 | Bacterial secretion system | 958009.26 | 630819.91 | 980807.52 | 532126.63 | 1167463.08 | 860597.29 |
